# Metabolic alterations in the bone tissues of aged osteoporotic mice

**DOI:** 10.1038/s41598-018-26322-7

**Published:** 2018-05-25

**Authors:** Miso Nam, Jeong-Eun Huh, Min-Sun Kim, Do Hyun Ryu, Jihyeong Park, Han-Sung Kim, Soo Young Lee, Geum-Sook Hwang

**Affiliations:** 1 Integrated Metabolomics Research Group, Western Seoul Center, Korea Basic Science Institute, Seoul, 03759 Republic of Korea; 2 Department of Chemistry, Sungkyunkwan University, Suwon, 16419 Republic of Korea; 3 Department of Life Science, Ewha Womans University, Seoul, 03760 Republic of Korea; 4 The Research Center for Cellular Homeostasis, Ewha Womans University, Seoul, 03760 Republic of Korea; 5 Department of Biomedical Engineering, College of Health Science, Institute of Medical Engineering, Yonsei University, Wonju, 220-710 Republic of Korea; 6 Department of Chemistry & Nanoscience, Ewha Womans University, Seoul, 03760 Republic of Korea

## Abstract

Age-related osteoporosis is characterized by reduced bone mineralization and reduced bone strength, which increases the risk of fractures. We examined metabolic changes associated with age-related bone loss by profiling lipids and polar metabolites in tibia and femur bone tissues from young (5 months old) and old (28 months old) male C57BL/6J mice using ultra-performance liquid chromatography quadrupole-time-of-flight mass spectrometry. Partial least-squares discriminant analysis showed clear differences in metabolite levels in bone tissues of young and old mice. We identified 93 lipid species, including free fatty acids, sphingolipids, phospholipids, and glycerolipids, that were significantly altered in bone tissues of old mice. In addition, the expression of 26 polar metabolites differed significantly in bone tissues of old mice and young mice. Specifically, uremic toxin metabolite levels (p-cresyl sulfate, hippuric acid, and indoxylsulfate) were higher in bone tissues of old mice than in young mice. The increase in p-cresyl sulfate, hippuric acid, and indoxylsulfate levels were determined using targeted analysis of plasma polar extracts to determine whether these metabolites could serve as potential osteoporosis biomarkers. This study demonstrates that LC-MS-based global profiling of lipid and polar metabolites can elucidate metabolic changes that occur during age-related bone loss and identify potential biomarkers of osteoporosis.

## Introduction

Osteoporosis, one of the most prevalent metabolic bone disorders, is characterized by low bone mass and microarchitectural deterioration of the bone structure due to an imbalance between bone resorption and bone formation^1,2^, thus patients with osteoporosis have fragile bones that are vulnerable to fractures. Osteoporosis is a major public health problem that primarily occurs in women after menopause and in aging individuals of both sexes^3^. The prevalence of osteoporosis and osteoporosis-related fractures increases exponentially after 50 years of age^4^, and advanced age has been shown to correlate with reductions in bone mass and bone strength^5,6^. As the aging population worldwide continues to grow, osteoporosis is becoming more prevalent and is leading to enormous medical costs for individuals and society because it causes physical pain and fractures of the hip, spine, and wrist^7,8^. Thus, it is necessary to understand the metabolism underlying age-related bone loss and osteoporosis and to identify potential biomarkers of osteoporosis to reduce the occurrence of osteoporosis and to prevent osteoporotic fractures.

Metabolomics, an important component of -omics technologies, which also includes genomics, transcriptomics, and proteomics, has been used to elucidate novel mechanisms of disease pathogenesis and to discover biomarkers for early disease diagnoses, and this has been done by comparing low molecular weight metabolite profiles under different conditions^9^. Many recent metabolomic studies have examined which metabolites and specific metabolic pathways are significantly altered during osteoporosis, and pathways involved in disturbance of energy metabolism, lipid metabolism, amino acid metabolism, gut microbiota, and kidney damage have been implicated in the development of osteoporosis^10–14^. These osteoporotic studies have mostly used ovariectomized (OVX) and glucocorticoid-induced animal models and samples from postmenopausal women^10^. However, a decrease of bone mass in the OVX mice and in postmenopausal women may also be associated with an estrogen deficiency, and is therefore not related only to changes of bone structure resulting from aging. To determine whether age-related bone loss occurs in the aging mouse model, we used the C57BL/6 J male mouse model, which is a widely used strain for metabolic studies. In addition, most of the metabolomic studies about osteoporosis analyzed metabolic alterations in biofluids, such as urine and blood (serum or plasma), whereas only a few metabolomic studies utilized bone tissues, which are the pathological source of osteoporosis. In our study, we performed a metabolomic analysis of tibia and femur bone tissues to observe more metabolic alterations associated with osteoporosis, thereby elucidating the molecular basis of osteoporosis.

Mass spectrometry (MS) in combination with chromatography is one of the most frequently used analytical techniques for global and targeted metabolomics^15–17^. Ultra-performance liquid chromatography-quadrupole-time-of-flight MS (UPLC-QTOF-MS) is a powerful tool for identifying metabolites because it provides high resolution and accurate masses for MS profiling and MS/MS experiments^17^. For quantitative analyses of targeted metabolites, UPLC-triple-quadrupole (TQ)-MS has become the analytical method of choice because it provides good sensitivity, reproducibility, and a broad dynamic range^18^. In this study, we were the first to profile lipid and polar metabolites and lipids in bone tissues of mice using UPLC-QTOF-MS and to quantify the metabolites that were significantly altered between young and old mice using UPLC-TQ-MS to determine the metabolic alterations and mechanisms that occur during age-related bone loss and to identify potential biomarkers of osteoporosis.

## Results

### Microcomputed tomography and microarchitectural parameters of tibia bone

**Figure 1:**
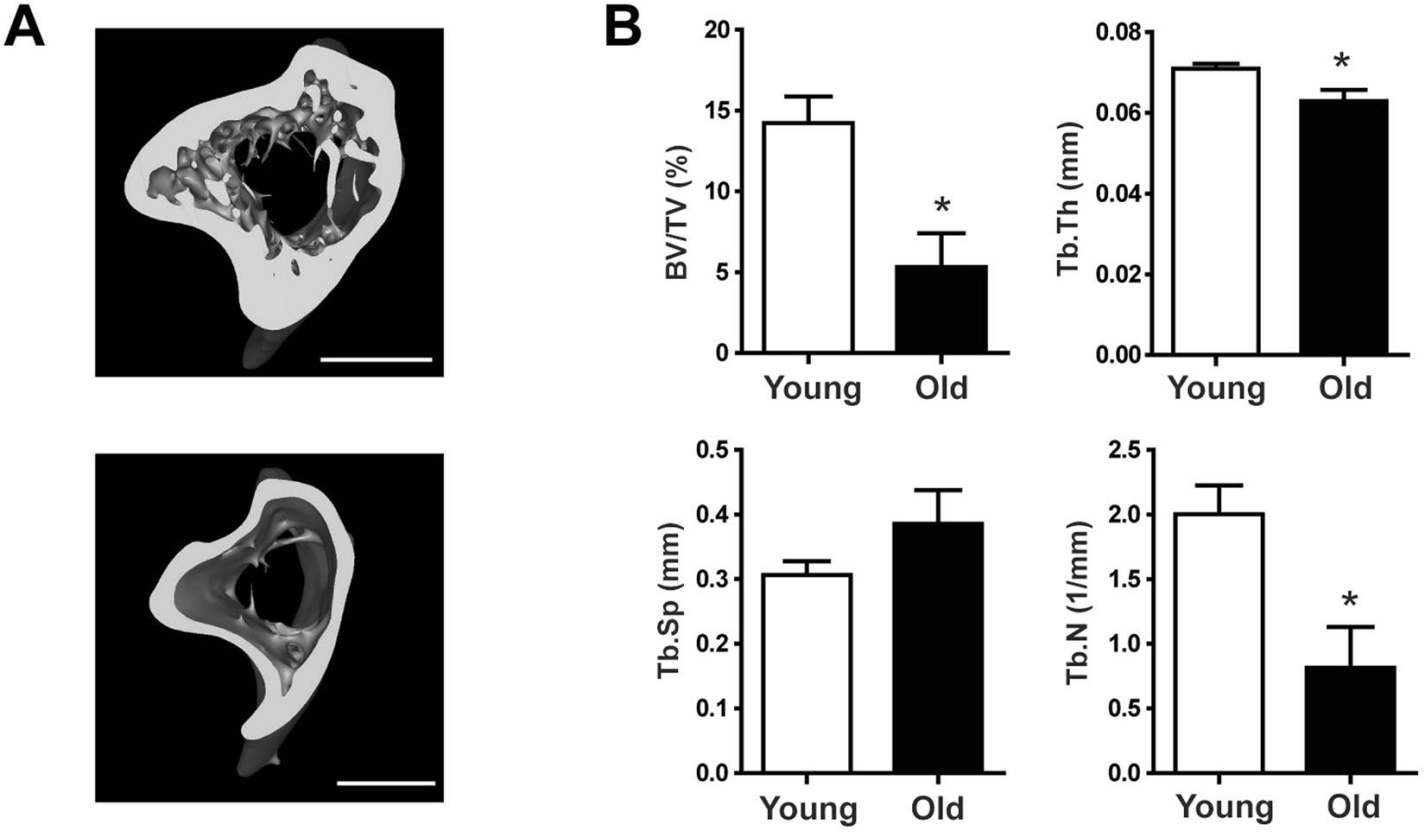
Bone mass was reduced in aged mice. (**A**) µCT images of tibias from young (upper panel) and old (lower panel) mice. (**B**) Microstructural parameters of tibias, including BV/TV, Tb.Th, Tb.Sp, and Tb.N in young and old mice. Results are expressed as mean ± SEM. *n* = 5 per group. Statistical differences between the parameters of bones from young and old mice were analyzed by the Mann-Whitney *U*-test. *p < 0.05. Scale bar, 1 mm.

To analyze metabolic changes in bone tissues associated with age-related bone loss, we used 5 month old (young) and 28 month old (old) male C57BL/6 J mice. We observed age-related bone loss by microcomputed tomography (μCT) of the tibia, which allowed us to measure microarchitectural parameters of the tibia, including tibia bone volume fraction (BV/TV), trabecular thickness (Tb.Th), trabecular separation (Tb.Sp), and trabecular number (Tb.N) (Fig. 1A). We clearly visualized reductions in bone volumes in the cancellous and cortical compartments and an increase in the medullary area in the bones of old mice. When compared with young mice, there was a 62.6% decrease in BV/TV, an 11.3% decrease in Tb.Th, and a 59.3% decrease in Tb.N (*p* < 0.05) in old mice (Fig. 1B). There was a 25.9% increase in Tb.Sp in old mice, but this increase was not statistically significant.

### Multivariate analysis of lipid and polar extracts from bone tissues

**Figure 2:**
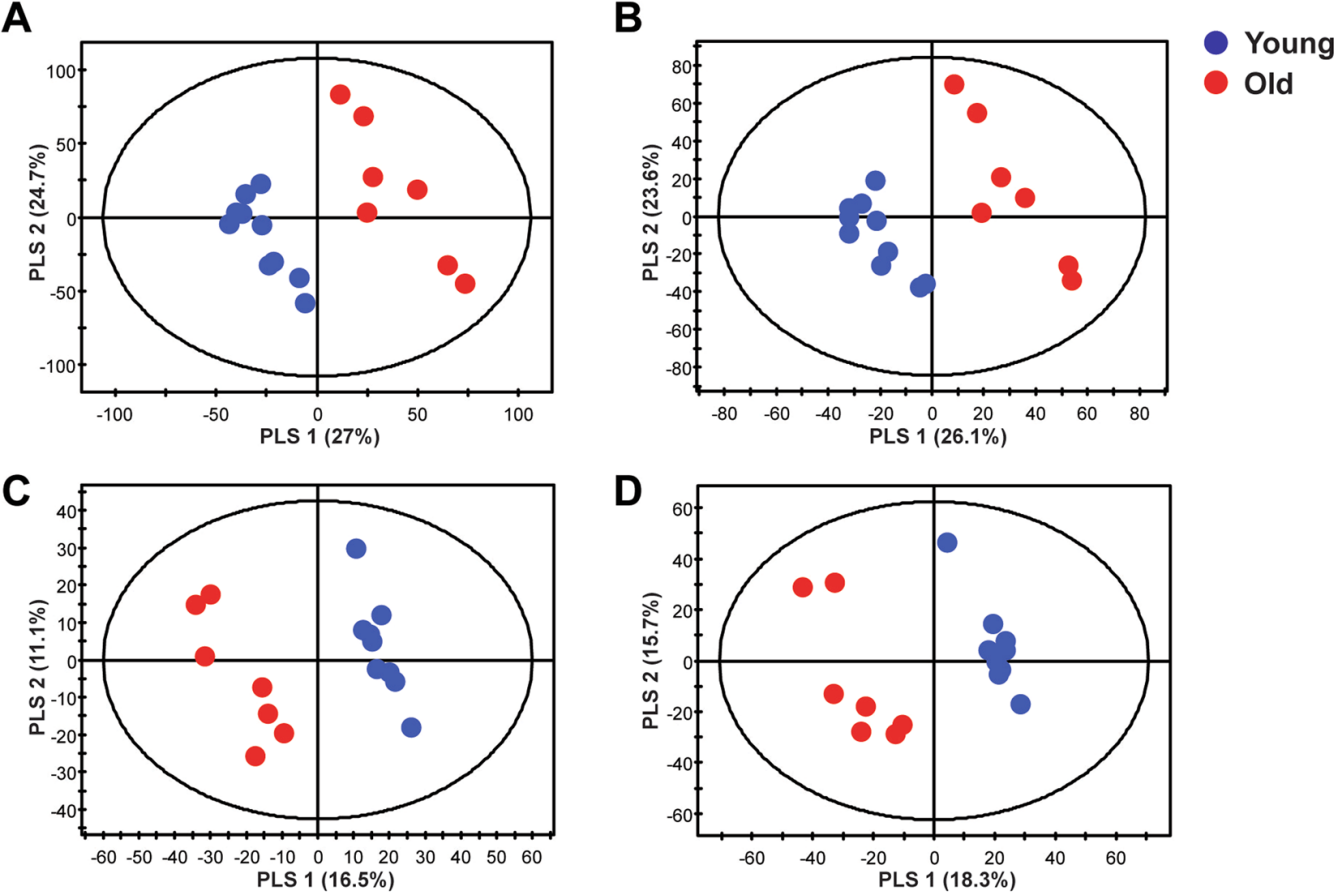
Multivariate statistical analysis of lipid and polar extracts of bone tissues from young and old mice. PLS-DA score plots from the spectra of (**A**) positive (R^2^X = 0.574, R^2^Y = 998, and Q^2^ = 0.915) and (**B**) negative (R^2^X = 0.549, R^2^Y = 995, and Q^2^ = 0.878) mode of UPLC-QTOF-MS in lipid metabolites and PLS-DA score plots from spectra of (**C**) positive (R^2^X = 0.276, R^2^Y = 0.969, and Q^2^ = 0.625) and (**D**) negative (R^2^X = 0.437, R^2^Y = 996, and Q^2^ = 0.918) mode of UPLC-QTOF-MS in polar metabolites.

To investigate differences in lipid and polar metabolites in bones from young and old mice, we performed global profiling in bone tissues. We obtained lipid and polar extracts from the bones of young and old mice and obtained a total of 6601 and 4550 aligned lipid metabolite features in UPLC-QTOF-MS positive and negative modes, respectively and obtained a total of 4313 and 4169 aligned polar metabolites features in UPLC-QTOF-MS positive and negative modes, respectively. Quality control (QC) samples were used to confirm the stability and reproducibility of the analysis. Equal amounts of all of the samples were pooled to generate a QC sample, and QC samples were analyzed prior to sample acquisition and after every five samples. The reproducibility of lipid and polar metabolites features was evaluated by coefficients of variation (CV) in QC samples and lipid and polar metabolites features showing large variations in QC samples were removed (CV > 20%). The lipid and polar metabolites features (CV < 20%) scaled to unit variance were analyzed by principal component analysis (PCA) and partial least-squares discriminant analysis (PLS-DA) to show the metabolic changes that occur during age-related bone loss. The PCA score plots, an unsupervised pattern recognition method, were derived from lipid-positive (Supplementary Fig. S1A) and lipid-negative (Supplementary Fig. S1B) modes and polar-positive (Supplementary Fig. S1C) and polar-negative (Supplementary Fig. S1D) modes. The QC samples clustered together on the PCA score plot indicating that our analysis was stable and reproducible. PCA score plots showed that metabolites from the bone tissues of young and old mice clustered separately. PLS-DA analysis was then used to further differentiate the metabolite profiles of the young and old mice. The PLS-DA score plots were derived from lipid-positive (Fig. 2A), lipid-negative (Fig. 2B), polar-positive (Fig. 2C), and polar-negative (Fig. 2D) modes. The PLS-DA models of the lipid MS spectra were generated using three components in positive mode (R^2^X = 0.574, R^2^Y = 998, and Q^2^ = 0.915) and three components in negative mode (R^2^X = 0.549, R^2^Y = 995, and Q^2^ = 0.878). The PLS-DA models of the polar MS spectra were generated using two components in positive mode (R^2^X = 0.276, R^2^Y = 0.969, and Q^2^ = 0.625) and three components in negative mode (R^2^X = 0.437, R^2^Y = 996, and Q^2^ = 0.918). The R^2^ and Q^2^ values indicated the quality of the PLS-DA model. R^2^ is defined as the proportion of variance in the data explained by the PLS-DA model and indicates goodness of fit, and Q^2^ is defined as the proportion of variance in the data predicted by the PLS-DA model and indicates predictability. The PLS-DA score plots show significant differences in polar and lipid metabolites in the bone tissues from young and old mice.

### Altered lipid and polar metabolites in old mice with osteoporosis

**Figure 3:**
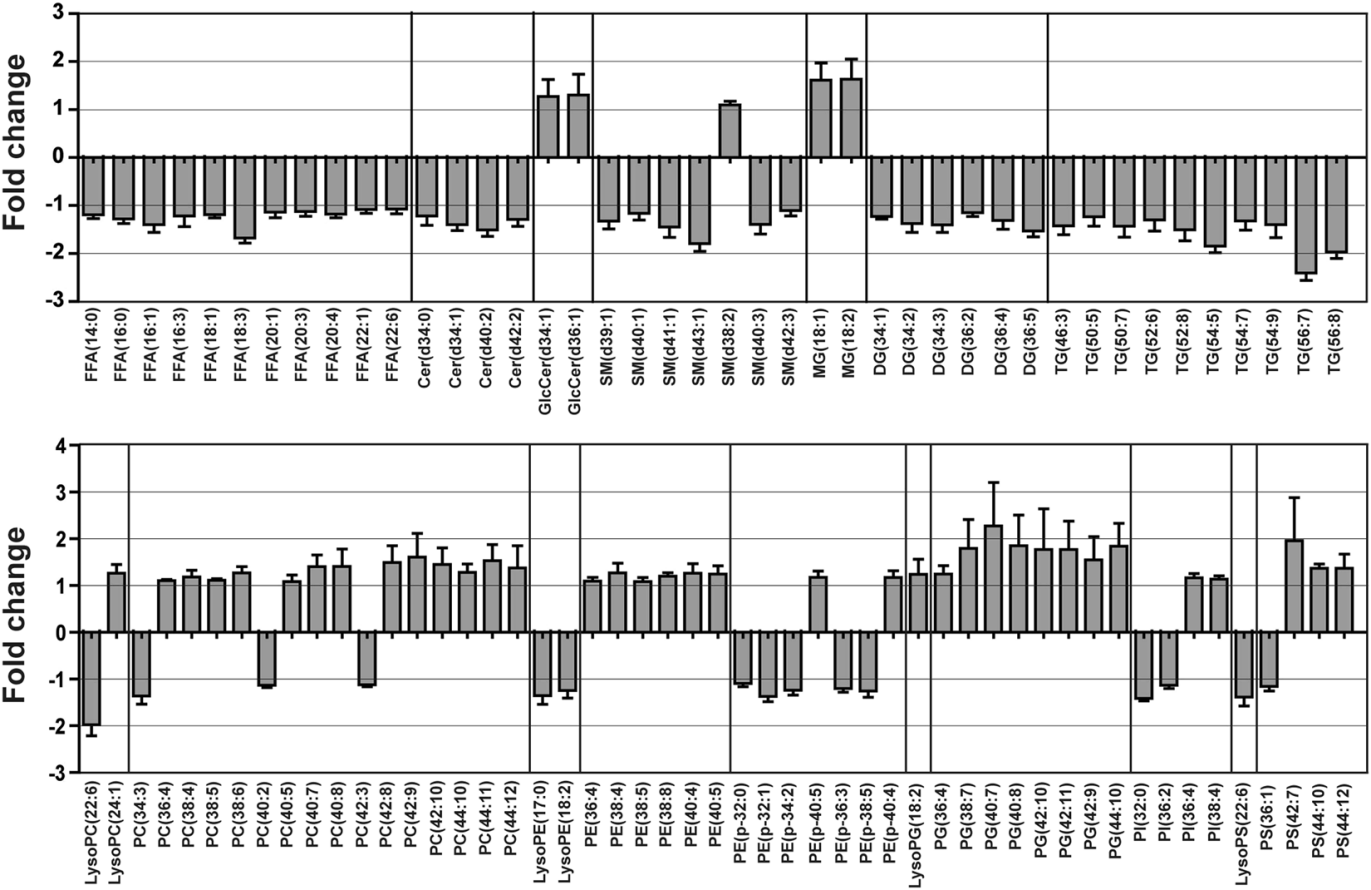
Levels of lipid species were altered in the bone tissues of old mice. Fold changes in the levels of lipid species found in the bone tissues of young and old mice.

To identify important lipid and polar metabolites associated with osteoporosis, the variable importance in the projection (VIP) and *p*-values of each variable were determined by PLS-DA and the Mann-Whitney *U*-test, respectively. All identified metabolites satisfied the criteria (i.e., VIP values > 1 and p-values < 0.05), and they were considered significant variables between young and old mice. Lipid and polar metabolites were tentatively identified using online databases and previously published references with mass tolerances of less than 10 ppm. The identities of the metabolites were confirmed by comparing their retention times and MS/MS spectral data to authentic standards. We identified 93 altered lipid species (VIP > 1, *p* < 0.05) (Fig. 3 and Supplementary Table S1) and 26 altered polar metabolites (VIP > 1, *p* < 0.05) (Supplementary Table S2). Analysis of lipid extracts of bone tissues from young and old mice showed that the levels of free fatty acids (FFA), sphingolipids [ceramide (Cer), sphingomyelin (SM)], diacylglycerol (DG), triacylglycerol (TG), and phosphatidylethanolamine-plasmalogen (PE-p) were significantly lower in the bone tissues of old mice than in the bone tissues of young mice. Glucosylceramide (GlcCer), monoacylglycerol (MG), and phospholipids [phosphatidylcholine (PC), phosphatidylethanolamine (PE), phosphatidylglycerol (PG), and phosphatidylserine (PS)] levels were higher in the bone tissues of old mice than in the bone tissues of young mice. We also examined a decrease in FFA in serum as well as a decrease in FFA in bone tissues (Supplementary Fig. S2).

**Figure 4:**
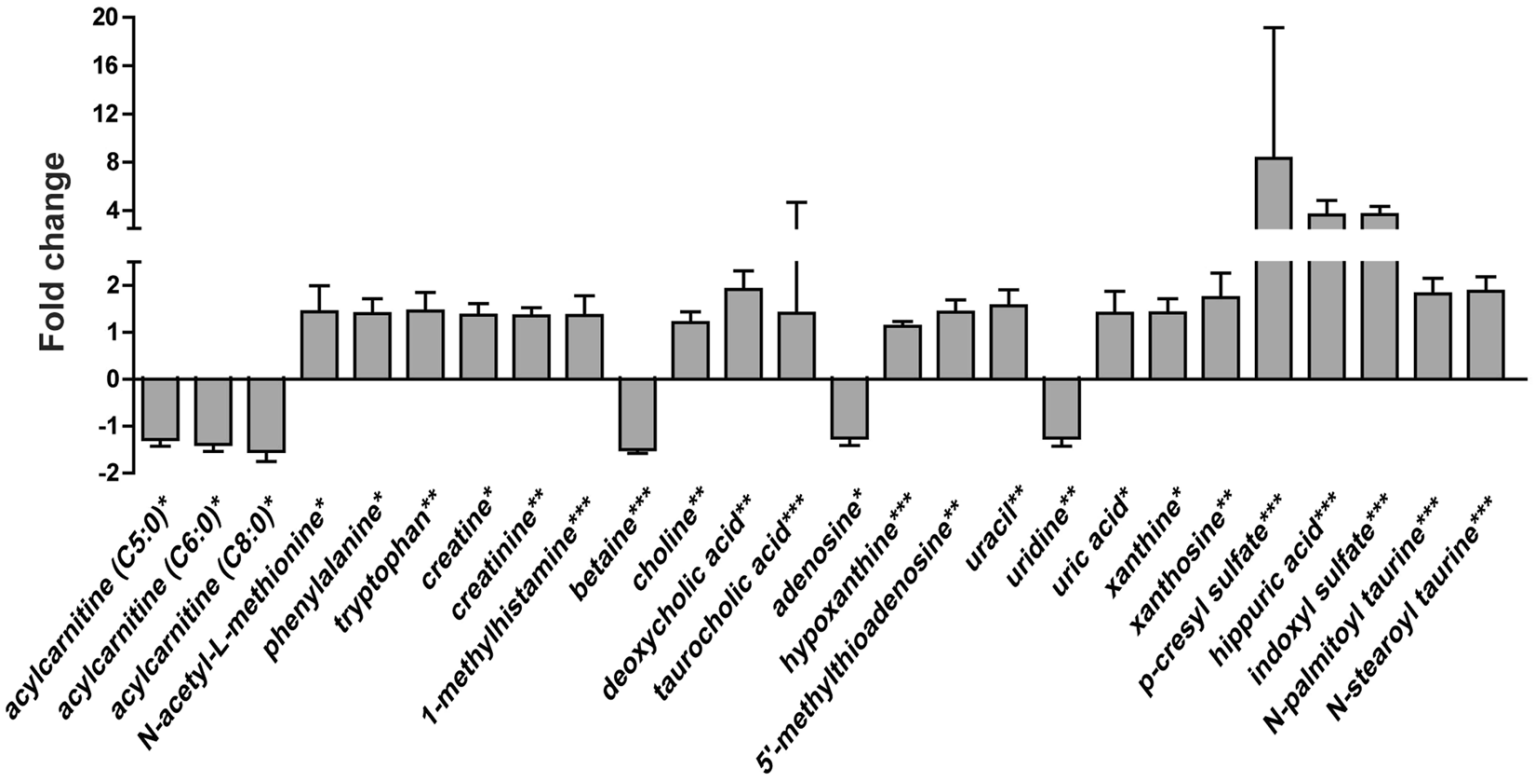
Polar metabolite levels were altered in the bone tissues of old mice. Fold changes in the levels of polar metabolites found in the bone tissues of young and old mice. Statistical differences between the polar metabolite levels in the bone tissues from young and old mice were determined using the Mann-Whitney *U*-test. *p < 0.05, **p < 0.01, ***p < 0.001.

Analysis of polar extracts showed that levels of choline, creatinine, niacinamide, 1-methylhistamine, creatine, adenine, hypoxanthine, xanthine, 5′-methylthioadenosine, taurocholic acid, p-cresol, uracil, phenylalanine, uric acid, hippuric acid, p-cresyl sulfate, N-acetyl-L-methionine, tryptophan, indoxylsulfuric acid, xanthosine, and deoxycholic acid were higher in the bone tissues of old mice than in the bone tissues of young mice, whereas levels of betaine, uridine, and acylcarnitine (C5:0, C6:0, and C8:0) were lower in the bone tissues of old mice than in the bone tissues of young mice (Fig. 4 and Supplementary Table S2).

### Quantification of metabolites in bone tissue and in plasma

**Figure 5:**
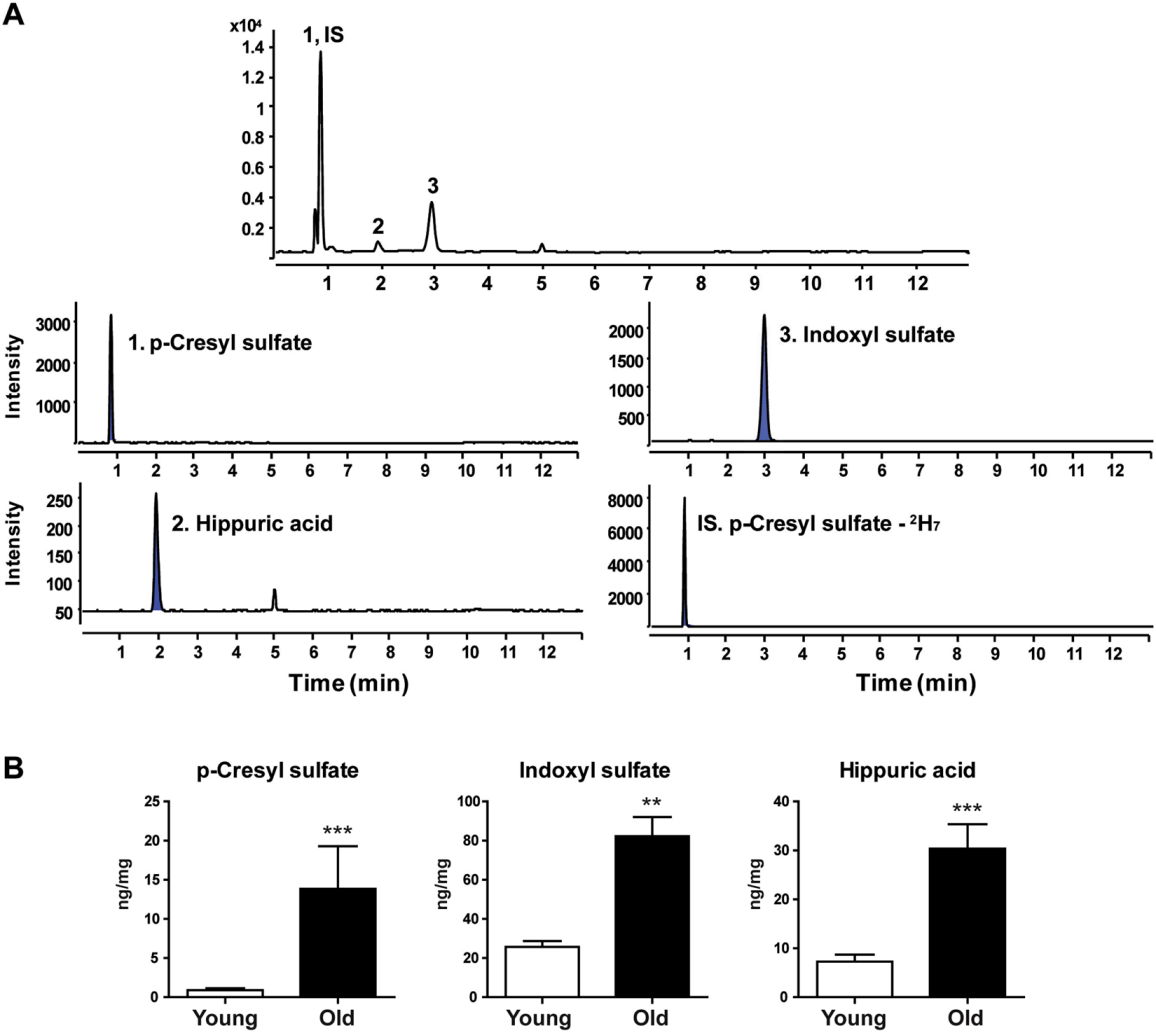
Quantification of uremic toxin metabolites in bone tissues using UPLC-TQ-MS. (**A**) Total ion chromatograms and MRM chromatograms of bone tissue extracts. (**B**) Concentrations of p-cresyl sulfate, indoxyl sulfate, and hippuric acid in bone tissue extracts. Results are expressed as mean ± SEM. Statistical differences in the levels of uremic toxin metabolites in bone tissues of young and old mice were determined using the Mann-Whitney *U*-test. **p < 0.01, ***p < 0.001.

**Figure 6:**
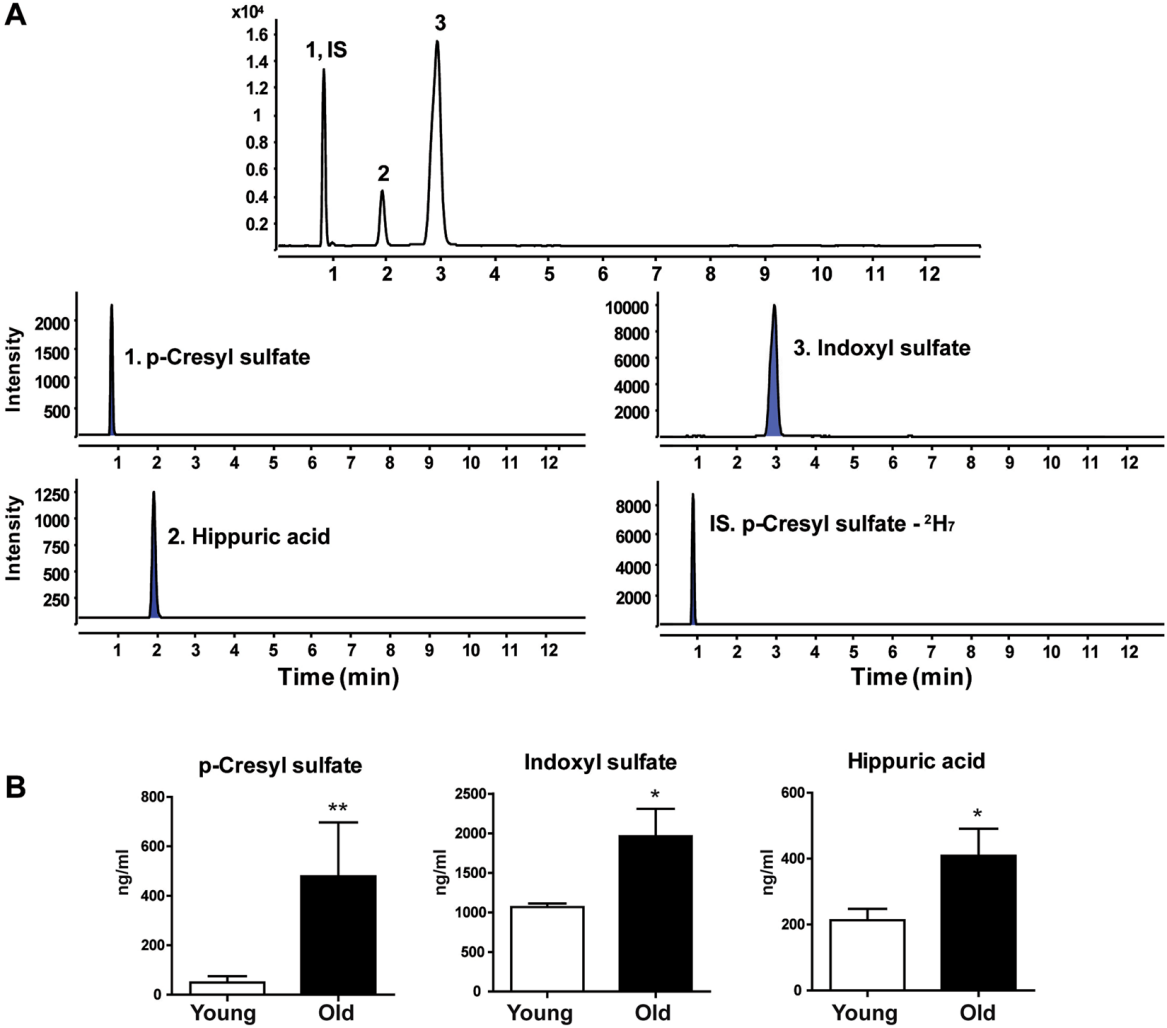
Quantification of uremic toxin metabolites in plasma using UPLC-TQ-MS. (**A**) Total ion chromatograms and MRM chromatograms of plasma extracts. (**B**) Concentrations of p-cresyl sulfate, indoxyl sulfate, and hippuric acid in plasma extracts. Results are expressed as mean ± SEM. Statistical differences in the levels of uremic toxin metabolites in plasma from young and old mice were determined using the Mann-Whitney *U*-test. *p < 0.05, **p < 0.01.

Based on global lipid and polar metabolite profiling data, we quantified the important metabolites associated with age-related bone loss using UPLC-TQ-MS. Because UPLC-QTOF-MS analysis showed that the levels of the uremic toxin metabolites p-cresyl sulfate, hippuric acid, and indoxylsulfate were much higher in the bone tissues of old mice than in the bone tissues of young mice, we conducted a targeted analysis of uremic toxin metabolites using UPLC-TQ-MS in the MRM mode because it is highly sensitive and selective. We also performed the targeted analysis of uremic toxin metabolites in polar extracts of plasma to determine if we could find potential biomarkers associated with age-related bone loss in plasma. Representative total ion chromatograms and MRM chromatograms of the targeted metabolites in bone tissues and plasma extracts are shown in Figs 5 and 6, respectively. For the best signal-to-noise ratios, MRM ion transitions were determined from MS/MS spectra, and p-cresyl sulfate-^2^H_7_ was used as the internal standard. The equations used to calculate the calibration curves and the linear correlation coefficients (*R*^2^) of the targeted metabolites in bone tissues and plasma are shown in Supplementary Tables S3 and S4. We found that the concentrations of p-cresyl sulfate, indoxyl sulfate, and hippuric acid were significantly higher in the bone tissues of old mice than in the bone tissues of young mice. Similarly, the concentrations of p-cresyl sulfate, indoxyl sulfate, and hippuric acid were significantly higher in the plasma extracts of old mice than in the plasma extracts of young mice. Finally, since bone marrow has a great influence on the change of the bone tissues, changes in uremic toxin metabolites in bone marrow were analyzed to observe if bone marrow shows alterations similar to those observed in bone tissues. Hippuric acid was not detected in bone marrow because the concentration was lower than the LOQ. The concentration of p-cresyl sulfate was significantly higher in the bone marrow of old mice compared to young mice. The concentration of indoxyl sulfate also increased in the old mice, but was not significant. (Supplementary Fig. S3).

## Discussion

In this study, we applied global profiling of bone tissues from young and old mice using UPLC-QTOF-MS analysis to identify the metabolic changes that occur during age-related bone loss, and then we quantified the metabolites that changed significantly using UPLC-TQ-MS analysis. It has been shown that male C57BL/6 J mice lose skeletal mass and bone architecture with age^19^. Also, it has been shown that bone tissue changes in aged male C57BL/6 J mice are similar to bone tissues change observed in aged humans^20^. Therefore, our male C57BL/6 J mouse model can be used to provide information on bone metabolism in age-related skeletal disorders in humans. Analysis of μCT images and microarchitectural parameters of the tibia showed that there were significant changes in the bones of old mice when compared to young mice and 28 months of age is old enough to examine age-related metabolic changes in the bone tissues of mice. UPLC-QTOF-MS analyses and PLS-DA score plots of lipid and polar extracts from bone tissues showed clear differences between young and old mice indicating that aging affects bone structure and lipid and polar metabolite levels in bone tissues.

Many previous studies have shown that osteoporosis is closely linked to changes in lipid levels. Yamaguchi *et al*. showed that plasma total cholesterol, low density lipoprotein cholesterol, high density lipoprotein cholesterol, and TG levels correlated with bone mineral density and that plasma lipid levels correlated with bone mass and bone fragility^21^. It has also been shown that lipid oxidation in bone increases with age and that this oxidation increases apoptosis of osteoblastic cells and inhibits bone morphogenetic protein-2-induced osteoblast differentiation^5^. However, no previous studies have used lipidomics technology to explore changes in lipid composition in bone tissues from mice with age-related osteoporosis. By applying multivariate statistical analysis and the Mann-Whitney *U*-test, we identified 93 lipids, including FFAs, sphingolipids (Cer and SM), phospholipids (PC, PE, PE-p, PG, and PS) and glycerolipids (MG. DG, and TG), that were present at significantly different levels in the bone tissues of young and old mice. These results showed that osteoporosis is closely related to lipid metabolism and further studies are needed to definitively determine the mechanisms of lipid metabolism that occur during age-related bone loss. The decreases in FFA levels in bone tissues and serum were examined. Dysregulation of acylcarnitine metabolism, which is involved in the translocation of fatty acyl chains from FFA through the mitochondrial membrane, was observed in aged bone tissues. Specifically, we found that medium-chain acylcarnitine (C5:0, C6:0, and C8:0) levels were lower in the bone tissues of old mice compared to that of young mice. This reduced production of medium-chain acylcarnitine indicated that FFA β-oxidation was inhibited by mitochondrial dysfunction, and that a disturbance in metabolic oxidation occurred^22^. Aging caused metabolic changes to shift glycolysis and decrease FFA β-oxidation, which resulted in increased lactate and reduced expression of genes involved in FFA β-oxidation^23^. Moreover, the presence of a glycolytic dominance in old bone tissues by mitochondrial dysfunction resulted in compromised energy metabolism, leading to the loss of bone and to a decline in bone formation^24^. In the future, further studies to prevent impaired oxidative metabolism and mitochondrial dysfunction could protect bone health during aging.

p-Cresyl sulfate, indoxyl sulfate, and hippuric acid, which are the most widely known uremic toxin metabolites containing phenyl groups, accumulated in the bone tissues of old mice and were the metabolites that differed the most in the bone tissues of old and young mice. In addition, higher concentrations of uremic toxin metabolites in the plasma and bone marrow of old mice were observed. The higher concentrations in the plasma of old mice suggest that they could be useful, non-invasive biomarkers of osteoporosis. The cross-validation of these uremic toxin metabolites in both bone tissue and plasma strengthens their potential as osteoporosis biomarkers. p-Cresyl sulfate and indoxyl sulfate originate from intestinal microbiota and these uremic toxin metabolites are representative microbiome-affected molecules known to be potentially harmful to the host^25^. It was found that plasma levels of p-cresyl sulfate, indoxyl sulfate, and hippuric acid in conventionally raised mice were increased compared with those of germ-free (GF) mice^26^. The elevated levels of these uremic toxin metabolites in bone and plasma in old mice may be associated with the changes of the composition of gut microbiota (GM) with age, and could lead to reduced bone mass. The GM affects both health and disease status, and has numerous functions, including alterations of host metabolism and immune status^27^. Recent studies suggested that the GM regulates bone mass via effects on the immune system^13,27,28^. Overgrowth of the GM was associated with bone loss and malabsorption, which can contribute to the occurrence of metabolic bone disease^29^. It was shown that bone mass was increased in the absence of GM in GF mice caused by a reduction in the expression of inflammatory cytokines such as IL-6 and TNFα in bone, and less osteoclastogenesis, compared to conventionally raised mice^13^. Therefore, imbalances in GM may have deleterious effects on bone metabolism. Age-related processes inevitably change the composition of the GM^30–32^. Altered GM composition with aging is linked to the host’s immune status and may affect bone metabolism. Furthermore, p-cresyl sulfate, indoxyl sulfate and hippuric acid play a significant role in inducing bone loss. p-Cresyl sulfate has been shown to significantly upregulate intracellular production of reactive oxygen species (ROS), which reduces proliferation and viability of osteoblastic cells and activates JNK and p38 mitogen-activated protein kinase pathways, which increase DNA fragmentation and reduce cAMP production in osteoblastic cells^33^. Similarly, indoxyl sulfate induced ROS production, reduced viability, and increased DNA fragmentation in osteoblastic cells^33^. In addition, Kim Y. found that indoxyl sulfate suppressed osteoblast-specific alkaline phosphatase activity and inhibited expression of mRNA type 1 collagen and osteonectin, which are only produced in differentiated osteoblasts indicating that indoxyl sulfate is a metabolite that is toxic to bone and inhibits osteoblast differentiation^34^. High blood indoxyl sulfate levels exacerbate low bone turnover, which is primarily characterized by an abnormally low bone formation rate^32^. Treatment with AST-120, which absorbs hydrophobic uremic substances, prevented accumulation of indoxyl sulfate in the blood and improved the rate of bone formation indicating that inhibition of indoxyl sulfate accumulation improves low-turnover bone progression^35^. Further, high levels of hippuric acid in the urine of OVX rats associated with postmenopausal osteoporosis^36^. Taken together, accumulation of p-cresyl sulfate, indoxyl sulfate, and hippuric acid in the bone tissues of old mice could be fatal to bone metabolism, including osteoblast differentiation and bone formation, and could lead to age-related osteoporosis.

We also observed that the levels of the essential aromatic amino acids tryptophan and phenylalanine were higher in the bone tissues of old mice than in the bone tissues of young mice. In addition, a negative association has been observed between elevated serum tryptophan levels and bone mass density in pre- and post-menopausal women^37^. Further, plasma tryptophan and phenylalanine levels were significantly higher in osteoporotic rats than in control rats, and this increase in tryptophan and phenylalanine was ameliorated by supplementation with an anti-osteoporosis medicinal herb^38,39^. Bile acid has also been shown to reduce osteoblast viability and bone formation^40^. In this study, we detected higher levels of deoxycholic acid and taurocholic acid in the bone tissues of old mice than in the bone tissues of young mice. This accumulation of bile acid in the bone tissues of old mice may have interrupted bone formation.

In summary, we used UPLC-QTOF-MS-based lipidomics and metabolomics to investigate lipid and polar metabolite changes that occur in bone tissues during age-related skeletal disorders. We identified 93 lipids and 26 polar metabolites associated with age-related osteoporosis by performing high-resolution MS/MS analysis, comparing authentic standards, and confirming them with the corresponding molecular formulas. We also quantified some of the metabolites using UPLC-TQ-MS in the MRM mode. This study demonstrated the utility of lipidomics and metabolomics to find potential biomarkers of osteoporosis that could be used to monitor related risk factors, diagnose osteoporosis, and to design strategies for the treatment of osteoporosis.

## Materials and Methods

### Animals

Eight-week-old male C57BL/6 J mice were obtained from The Jackson Laboratory (Bar Harbor ME, USA) and maintained in an animal room until they reached 28 months of age. Animals were maintained in a controlled environment with a constant temperature of 24 °C, with fixed artificial light (12 h light-dark cycle). All of the experiments were approved by the Institutional Animal Care and Use Committee of Ewha Laboratory Animal Genomics Center and were carried out in accordance with the approved guidelines. Heparinized plasma and tibia and femur bone tissues, which the bone marrows flushed out using 1 ml PBS with a syringe and 25-gauge needle, were frozen in liquid nitrogen for further metabolomics analyses.

### Microcomputed tomography (μCT)

To evaluate bone mass and architecture by μCT, mouse tibias were fixed and scanned using a Skyscan 1076 *in vivo* μCT scanner (Bruker Corporation, Karlsruhe, Germany). Three-dimensional images obtained by CT-An (Skyscan) were analyzed to measure structural parameters, including BV/TV, Tb.Th, Tb.Sp, and Tb.N.

### Bone tissue, bone marrow and plasma extractions

Metabolites were extracted from bone and plasma. To extract metabolites from bone tissues, samples were freeze-dried for 3 h. Freeze-dried bone was transferred to a 1.5-ml tube containing 2.8 mm zirconium oxide beads and homogenized twice at 6000 rpm for 20 s using a Precellys 24 tissue grinder (Bertin Technologies, Ampère Montigny-le-Bretonneux, France). Nine-hundred μL of a chloroform:methanol mixture (2:1 v/v) was added to the tube and the sample was re-homogenized using the initial homogenization conditions. After homogenization, 180 μL of water was added to the sample and it was vortexed for 1 min. The sample was then incubated at 4 °C for 10 min and centrifuged at 13,000 rpm at 4 °C for 10 min. The aqueous phase supernatant was transferred to a new 1.5-ml tube and was vacuum dried. The organic phase was transferred to a new 1.5-ml tube and dried under a stream of nitrogen. Bone marrows with PBS flushed from bone tissue were dried under a stream of nitrogen and extracted in the same way as the bone tissues. The extracts were then stored at −80 °C.

To extract metabolites from plasma, 500 μL of a chloroform:methanol mixture (2:1 v/v) was added to 50 μL of plasma and the sample was vortexed for 30 s. Then, 100 μL of water was added and the sample was vortexed for 30 s. After vortexing, the sample was incubated at 4 °C for 10 min and centrifuged at 13,000 rpm at 4 °C for 10 min. The aqueous supernatant was transferred to a new 1.5-ml tube and vacuum dried. The organic phase was transferred to a new 1.5-ml tube and dried under a stream of nitrogen. The extracts were then stored at −80 °C.

### UPLC-QTOF-MS analysis

For the UPLC-QTOF-MS analysis, the polar bone extract (aqueous phase) was diluted with 300 μL of 75% (v/v) acetonitrile and the lipid bone extract (organic phase) was diluted with an isopropanol:acetonitrile:water mixture (2:1:1 v/v/v). The UPLC-QTOF-MS analysis was performed on an ACQUITY UPLC system (Waters, Milford, MA, USA) with a triple TOF™ 5600 mass spectrometer equipped with an electrospray ionization (ESI) source (AB Sciex, Concord, ON, Canada). Chromatographic separation of polar extracts was carried out at 35 °C on a ZIC®-HILIC column (2.1 mm × 100 mm, 3.5 μm; SeQuant) with a binary gradient at a flow rate of 0.4 ml/min. The mobile phases consisted of 10 mM ammonium acetate and 0.1% formic acid in water:acetonitrile (5:95 v/v, solvent A) and 10 mM ammonium acetate and 0.1% formic acid in water:acetonitrile (50:50 v/v, solvent B). The steps of the gradient profile used to equilibrate the initial gradient for subsequent runs were 1% B from 0–2 min, 1–55% B from 2–8 min, 55–99% B from 8–9 min, 99% B from 9–11 min, 99–1% B from 11–11.1 min, and 99% B from 11.1–15 min. The total run-time for each injection was 15 min and the injection volume was 5 μL. Chromatographic separation of lipid extracts was carried out at 35 °C on an Acquity UPLC BEH C18 column (2.1 mm × 100 mm, 1.7 μm; Waters) with a binary gradient at a flow rate of 0.35 ml/min. The mobile phases consisted of 10 mM ammonium acetate in water:acetonitrile (60:40 v/v, solvent A) and 10 mM ammonium acetate in isopropanol:acetonitrile (90:10 v/v, solvent B). The steps of the gradient profile used to equilibrate the initial gradient for subsequent runs were 40–65% B from 0–5 min, 65–70% B from 5–12 min, 70–99% B from 12–15 min, 99% B from 15–17 min, 99–40% B from 17–17.1 min, and 40% B from 17.1–20 min. The total run-time for each injection was 20 min and the injection volume was 5 μL.

The mass spectrometer was operated in positive and negative ion modes and data were acquired in the mass range of 50–1000 *m/z* for analysis of polar extracts and from 100–1500 *m/z* for analysis of lipid extracts. Total ion chromatograms were acquired using the following operation parameters: capillary voltages of +4,500 V and −4,500 V for the positive and negative modes, a nebulizer pressure of 50 psi, a drying gas pressure of 60 psi, a curtain gas pressure of 30 psi, a source temperature of 500 °C, a declustering potential of ±90 eV, a collision energy of ±10 eV for single MS, and a collision energy of ±35 eV for MS/MS of polar extracts and ±45 eV for MS/MS of lipid extracts. Data from MS/MS analyses were acquired by automatic fragmentation in which the five most intense mass peaks were fragmented. Mass accuracy was maintained by use of an automated calibrant delivery system interfaced to the second inlet of the DuoSpray source. Equal amounts of all of the samples were pooled to generate a QC sample. QC samples were analyzed prior to sample acquisition and after every five samples to monitor the stability and reproducibility of the analytical system.

### Data processing of UPLC-QTOF-MS spectra

Spectral data were analyzed with MarkerView^TM^ (AB Sciex) to find peaks, perform alignments, and to generate peak tables of *m/z* and retention times for samples. Batch normalization^41^ and total ion normalization were employed to remove systematic variations between experiments from the samples. Batch normalization was performed using R software (version 2.13.1). Polar metabolites were identified by comparing experimental MS and MS/MS data with various online databases, including metlin (metlin.scrpps.edu), HMDB (www.hmdb.ca), and MassBank (www.massbank.jp), and were confirmed by comparing experimental data with the retention times and MS/MS spectra of authentic standard samples. Lipids were identified by comparing experimental data with authentic references and various lipid metabolite databases, including lipidmap (www.lipidmaps.org), metlin (metlin.scripps.edu), human metabolome (www.hmdb.ca), and our in-house library. Isotope patterns and fragment patterns (MS/MS spectra) were matched to identify lipid metabolites.

### UPLC-TQ-MS analysis for targeted profiling

To quantify levels of uremic toxins and related metabolites, targeted profiling was performed on Agilent 1290 Infinity LC and Agilent 6490 Triple Quadrupole MS systems equipped with an Agilent Jet Stream ESI source (Agilent Technologies, Palo Alto, CA, USA). MassHunter Workstation (Ver B.06.00, Agilent Technologies) software was used for data acquisition and analysis. Chromatographic separation was performed using a ZIC®-HILIC column (2.1 mm × 100 mm, 3.5 μm; SeQuant). The flow rate and injection volume were set at 0.4 ml/min and 1 μl, respectively. Mobile phase A consisted of 10 mM ammonium acetate and 0.1% formic acid in water:acetonitrile (5:95 v/v), and phase B consisted of 10 mM ammonium acetate and 0.1% formic acid in water:acetonitrile (50:50 v/v). The linear gradient used for elution and to equilibrate the initial gradient for subsequent runs was 1% B from 0–2 min, 1–55% B from 2–6 min, 55–99% B from 6–7 min, 99% B from 7–9 min, 99–1% B from 9–9.1 min, and 99% B from 9.1–13 min. Quantification was performed in the MRM mode and the optimal conditions for each metabolite were determined by flow injection of individual standards (100 ng/mL in 75% acetonitrile) into the ESI source in the negative ion mode. p-Cresyl sulfate-^2^H_7_ was used as an internal standard. Compound retention times and MRM transitions are summarized in Supplementary Table S5.

### Statistical analysis

Multivariate statistical analysis was performed using SIMCA-P^+^ software (version 12.0, Umetrics, Umeå, Sweden). PCA is an unsupervised method that analyzes samples without information regarding the samples and PLS-DA is a supervised analysis that uses class information to maximize the separation between classes and minimize the distance between intragroup clustering. Prior to PCA and PLS-DA, all of the variables obtained from the UPLC-QTOF-MS data sets were scaled to unit variance. Statistical analysis was performed using the Mann-Whitney *U*-test with the Statistical Package for the Social Sciences software version 21.0 (SPSS Inc., Chicago, IL, USA). A two-tailed *p*-value < 0.05 was considered statistically significant.

### Measurement of serum FFA

Blood samples were collected from abdominal aorta and centrifuged to isolate serum. FFA levels were measured using a Hitachi 7600 clinical analyzer (Hitachi, Tokyo, Japan).

## Electronic supplementary material

Supplementary Information
