# Supplementary material for: Metabolic alterations in the bone tissues of aged osteoporotic mice
Source: Sci Rep. 2018 May 25;8:8127. doi: 10.1038/s41598-018-26322-7 (PMC5970270; doi:10.1038/s41598-018-26322-7)
Supplement: Supplementary file 1 — Supplementary Information [file 41598_2018_26322_MOESM1_ESM.docx]

**Metabolic alterations in the bone tissues of aged osteoporotic mice**

Miso Nam^1,2,*^, Jeong-Eun Huh^3,4,*^, Min-Sun Kim^1^, Do Hyun Ryu^2^, Jihyeong Park^5^, Han-Sung Kim^5^, Soo Young Lee^3,4,§^, and Geum-Sook Hwang^1,6, §^

^1^Integrated Metabolomics Research Group, Western Seoul Center, Korea Basic Science Institute, Seoul 03759, Republic of Korea.

^2^Department of Chemistry, Sungkyunkwan University, Suwon 16419, Republic of Korea.

^3^Department of Life Science, Ewha Womans University, Seoul 03760, Republic of Korea.

^4^The Research Center for Cellular Homeostasis, Ewha Womans University, Seoul 03760, Republic of Korea.

^5^Department of Biomedical Engineering, College of Health Science, Institute of Medical Engineering, Yonsei University, Wonju 220-710, Republic of Korea.

^6^Department of Chemistry & Nanoscience, Ewha Womans University, Seoul 03760, Republic of Korea.

*These authors contributed equally to this work.

^§^**Corresponding author**

Geum-Sook Hwang: Korea Basic Science Institute, Seoul 03759, Republic of Korea. Phone: +82-2-6908-6200. Fax: +82-2-6908-6239. E-mail: gshwang@kbsi.re.kr

Soo Young Lee: Department of Life Science, Ewha Womans University, Seoul 03760, Republic of Korea. Phone: +82-2-3277-3770. Fax: +82-2-3277-3762. E-mail: leesy@ewha.ac.kr

**Supplementary Table S1.** Significantly altered lipids in old mice.

| peak no. | Compound | RT (min) | *m/z* | Difference (ppm) | Ionization mode | Molecular formula | Fold change (old/young) | VIP value | *p*-value |
| --- | --- | --- | --- | --- | --- | --- | --- | --- | --- |
| 1 | FFA(14:0) | 2.12 | 227.202 | 1 | - | C14H27O2- | 0.84 | 1.48 | 0.005 |
| 2 | FFA(16:0) | 2.93 | 255.2319 | 4 | - | C16H31O2- | 0.78 | 1.73 | 0 |
| 3 | FFA(16:1) | 2.28 | 253.2169 | 1 | - | C16H29O2- | 0.72 | 1.32 | 0.01 |
| 4 | FFA(16:3) | 1.51 | 249.1855 | 1 | - | C16H25O2- | 0.82 | 1.02 | 0.043 |
| 5 | FFA(18:1) | 3.25 | 281.2482 | 1 | - | C18H33O2- | 0.84 | 1.68 | 0.002 |
| 6 | FFA(18:3) | 2.02 | 277.2162 | 3 | - | C18H29O2- | 0.60 | 1.94 | 0 |
| 7 | FFA(20:1) | 4.26 | 309.2784 | 4 | - | C20H37O2- | 0.88 | 1.33 | 0.014 |
| 8 | FFA(20:3) | 2.85 | 305.2472 | 4 | - | C20H33O2- | 0.89 | 1.33 | 0.01 |
| 9 | FFA(20:4) | 2.29 | 303.2318 | 3 | - | C20H31O2- | 0.85 | 1.33 | 0.019 |
| 10 | FFA(22:1) | 5.29 | 337.3081 | 9 | - | C22H41O2- | 0.92 | 1.17 | 0.043 |
| 11 | FFA(22:6) | 2.19 | 327.2304 | 7 | - | C22H31O2- | 0.93 | 1.16 | 0.043 |
| 12 | Cer(d34:0) | 10.71 | 540.5392 | 7 | + | C34H70NO3+ | 0.82 | 1.14 | 0.033 |
| 13 | Cer(d34:1) | 10.06 | 538.5164 | 5 | + | C34H68NO3+ | 0.72 | 1.49 | 0.005 |
| 14 | Cer(d40:2) | 13.71 | 620.593 | 7 | + | C40H78NO3+ | 0.66 | 1.64 | 0 |
| 15 | Cer(d42:2) | 14.34 | 648.6246 | 6 | + | C42H82NO3+ | 0.78 | 1.52 | 0.002 |
| 16 | GlcCer(d34:1) | 8.96 | 700.5703 | 2 | + | C40H78NO8+ | 1.27 | 1.33 | 0.033 |
| 17 | GlcCer(d36:1) | 10.69 | 728.5995 | 5 | + | C42H82NO8+ | 1.30 | 1.29 | 0.003 |
| 18 | SM(d39:1) | 13.17 | 773.6484 | 6 | + | C44H90N2O6P+ | 0.76 | 1.26 | 0.043 |
| 19 | SM(d40:1) | 13.69 | 809.6482 | 3 | + | C45H91N2O6PNa+ | 0.86 | 1.18 | 0.025 |
| 20 | SM(d41:1) | 14.04 | 801.6873 | 3 | + | C46H94N2O6P+ | 0.69 | 1.29 | 0.025 |
| 21 | SM(d43:1) | 14.59 | 829.712 | 4 | + | C48H98N2O6P+ | 0.56 | 1.64 | 0.001 |
| 22 | SM(d38:2) | 10.31 | 757.6159 | 7 | + | C43H86N2O6P+ | 1.10 | 1.18 | 0.033 |
| 23 | SM(d40:3) | 9.23 | 805.6196 | 0 | + | C45H87N2O6PNa+ | 0.72 | 1.23 | 0.033 |
| 24 | SM(d42:3) | 12.22 | 833.6445 | 7 | + | C47H91N2O6PNa+ | 0.91 | 1.19 | 0.043 |
| 25 | MG(18:1) | 6.72 | 339.2891 | 2 | + | C21H41O5+ | 1.61 | 1.52 | 0.01 |
| 26 | MG(18:2) | 6.07 | 337.2748 | 1 | + | C21H39O5+ | 1.63 | 1.55 | 0.003 |
| 27 | DG(34:1) | 8.09 | 577.5172 | 4 | + | C37H71O6+ | 0.81 | 1.61 | 0.003 |
| 28 | DG(34:2) | 7.03 | 575.502 | 3 | + | C37H69O6+ | 0.73 | 1.18 | 0.025 |
| 29 | DG(34:3) | 9.97 | 608.5221 | 4 | + | C37H70NO5+ | 0.71 | 1.37 | 0.007 |
| 30 | DG(36:2) | 8.42 | 603.5321 | 5 | + | C39H73O6+ | 0.87 | 1.38 | 0.014 |
| 31 | DG(36:4) | 10.37 | 634.5371 | 5 | + | C39H72NO5+ | 0.77 | 1.56 | 0 |
| 32 | DG(36:5) | 9.14 | 632.5237 | 1 | + | C39H70NO5+ | 0.66 | 1.80 | 0 |
| 33 | TG(46:3) | 14.97 | 790.6885 | 4 | + | C49H92NO6+ | 0.70 | 1.23 | 0.014 |
| 34 | TG(50:5) | 15.02 | 842.7215 | 2 | + | C53H96NO6+ | 0.81 | 1.33 | 0.002 |
| 35 | TG(50:7) | 14.99 | 821.6582 | 8 | + | C53H89O6+ | 0.70 | 1.28 | 0.002 |
| 36 | TG(52:6) | 15.06 | 868.7353 | 4 | + | C55H98NO6+ | 0.77 | 1.25 | 0.025 |
| 37 | TG(52:8) | 15.05 | 847.6752 | 6 | + | C55H91O6+ | 0.66 | 1.30 | 0.014 |
| 38 | TG(54:5) | 14.78 | 881.7588 | 0 | + | C57H101O6+ | 0.54 | 1.79 | 0 |
| 39 | TG(54:7) | 15.09 | 894.7501 | 4 | + | C57H100NO6+ | 0.76 | 1.29 | 0.01 |
| 40 | TG(54:9) | 15.07 | 873.6905 | 7 | + | C57H93O6+ | 0.72 | 1.16 | 0.043 |
| 41 | TG(56:7) | 14.45 | 905.7564 | 3 | + | C59H101O6+ | 0.42 | 1.78 | 0.001 |
| 42 | TG(56:8) | 13.96 | 920.7617 | 9 | + | C59H102O6N+ | 0.51 | 1.77 | 0 |
| 43 | LysoPC(22:6) | 1.94 | 568.3372 | 4 | + | C30H51NO7P+ | 0.50 | 1.04 | 0.01 |
| 44 | LysoPC(24:1) | 5.76 | 606.4464 | 4 | + | C32H65NO7P+ | 1.26 | 1.16 | 0.033 |
| 45 | PC(34:3) | 5.6 | 756.5549 | 1 | + | C42H79NO8P+ | 0.73 | 1.19 | 0.025 |
| 46 | PC(36:4) | 8.69 | 782.5671 | 2 | + | C44H81NO8P+ | 1.10 | 1.73 | 0 |
| 47 | PC(38:4) | 10.36 | 810.598 | 3 | + | C46H85NO8P+ | 1.18 | 1.61 | 0.001 |
| 48 | PC(38:5) | 8.77 | 808.583 | 2 | + | C46H83NO8P+ | 1.11 | 1.87 | 0 |
| 49 | PC(38:6) | 7.7 | 806.5678 | 2 | + | C46H81NO8P+ | 1.27 | 1.47 | 0.005 |
| 50 | PC(40:2) | 13.75 | 842.6577 | 6 | + | C48H93NO8P+ | 0.88 | 1.44 | 0.005 |
| 51 | PC(40:5) | 10.42 | 836.6143 | 2 | + | C48H87NO8P+ | 1.08 | 1.45 | 0 |
| 52 | PC(40:7) | 8 | 832.5835 | 1 | + | C48H83NO8P+ | 1.40 | 1.20 | 0.025 |
| 53 | PC(40:8) | 7.21 | 830.5654 | 4 | + | C48H81NO8P+ | 1.41 | 1.02 | 0.043 |
| 54 | PC(42:3) | 13.86 | 868.68 | 1 | + | C50H95NO8P+ | 0.89 | 1.25 | 0.043 |
| 55 | PC(42:8) | 8.43 | 858.597 | 4 | + | C50H85NO8P+ | 1.49 | 1.47 | 0.002 |
| 56 | PC(42:9) | 7.49 | 856.5787 | 7 | + | C50H83NO8P+ | 1.61 | 1.49 | 0.005 |
| 57 | PC(42:10) | 7.12 | 854.5664 | 3 | + | C50H81NO8P+ | 1.45 | 1.32 | 0.014 |
| 58 | PC(44:10) | 7.94 | 882.5995 | 1 | + | C52H85NO8P+ | 1.28 | 1.04 | 0.043 |
| 59 | PC(44:11) | 7.22 | 880.5823 | 3 | + | C52H83NO8P+ | 1.53 | 1.57 | 0.003 |
| 60 | PC(44:12) | 6.86 | 878.5661 | 3 | + | C52H81NO8P+ | 1.37 | 1.27 | 0.033 |
| 61 | LysoPE(17:0) | 3.37 | 466.2903 | 7 | - | C22H47NO7P- | 0.74 | 1.24 | 0.043 |
| 62 | LysoPE(18:2) | 2.2 | 478.289 | 7 | + | C23H45NO7P+ | 0.80 | 1.17 | 0.025 |
| 63 | PE(36:4) | 9 | 740.5203 | 2 | + | C41H75NO8P+ | 1.09 | 1.16 | 0.025 |
| 64 | PE(38:4) | 10.71 | 768.5485 | 6 | + | C43H79NO8P+ | 1.27 | 1.46 | 0.014 |
| 65 | PE(38:5) | 9.07 | 766.5342 | 5 | + | C43H77NO8P+ | 1.08 | 1.10 | 0.033 |
| 66 | PE(38:8) | 6.63 | 742.4768 | 5 | + | C43H71NO9P+ | 1.20 | 1.49 | 0.002 |
| 67 | PE(40:4) | 12.12 | 796.5842 | 1 | + | C45H83NO8P+ | 1.26 | 1.31 | 0.019 |
| 68 | PE(40:5) | 10.81 | 794.5659 | 4 | + | C45H81NO8P+ | 1.25 | 1.36 | 0.007 |
| 69 | PE(P-32:0) | 11.38 | 676.5234 | 6 | + | C37H75NO7P+ | 0.91 | 1.26 | 0.033 |
| 70 | PE(P-32:1) | 9.63 | 674.5102 | 2 | + | C37H73NO7P+ | 0.73 | 1.67 | 0.001 |
| 71 | PE(P-34:2) | 9.79 | 700.5258 | 2 | + | C39H75NO7P+ | 0.80 | 1.66 | 0.005 |
| 72 | PE(P-40:5) | 11.7 | 776.5552 | 6 | - | C45H79NO7P- | 1.17 | 1.35 | 0.014 |
| 73 | PE(P-36:3) | 10.07 | 726.5389 | 5 | + | C41H77NO7P+ | 0.83 | 1.75 | 0.001 |
| 74 | PE(P-38:5) | 9.6 | 750.5392 | 5 | + | C43H77NO7P+ | 0.80 | 1.56 | 0.01 |
| 75 | PE(P-40:4) | 13.52 | 780.5894 | 0 | + | C45H83NO7P+ | 1.17 | 1.33 | 0.033 |
| 76 | LysoPG(18:2) | 1.72 | 507.2738 | 1 | - | C24H44O9P- | 1.24 | 1.13 | 0.025 |
| 77 | PG(36:4) | 6.07 | 788.5413 | 2 | + | C42H79NO10P+ | 1.25 | 1.48 | 0.019 |
| 78 | PG(38:7) | 5.5 | 791.4848 | 2 | - | C44H72O10P- | 1.79 | 1.42 | 0.025 |
| 79 | PG(40:7) | 6.03 | 819.5144 | 4 | - | C46H76O10P- | 2.27 | 1.57 | 0.001 |
| 80 | PG(40:8) | 5.81 | 817.4993 | 3 | - | C46H74O10P- | 1.85 | 1.51 | 0.007 |
| 81 | PG(42:10) | 5.71 | 841.4972 | 6 | - | C48H74O10P- | 1.77 | 1.30 | 0.019 |
| 82 | PG(42:11) | 5.24 | 839.4847 | 2 | - | C48H72O10P- | 1.77 | 1.50 | 0.025 |
| 83 | PG(42:9) | 5.94 | 862.5572 | 2 | + | C48H81NO10P+ | 1.55 | 1.44 | 0.025 |
| 84 | PG(44:10) | 5.98 | 888.5757 | 0 | + | C50H83NO10P+ | 1.84 | 1.67 | 0 |
| 85 | PI(32:0) | 7.99 | 809.5145 | 4 | - | C41H78O13P- | 0.71 | 1.87 | 0 |
| 86 | PI(36:2) | 8.44 | 861.5416 | 9 | - | C45H82O13P- | 0.88 | 1.46 | 0.005 |
| 87 | PI(36:4) | 7.08 | 876.5593 | 0 | + | C45H83NO13P+ | 1.16 | 1.45 | 0.014 |
| 88 | PI(38:4) | 8.25 | 885.5451 | 5 | - | C47H82O13P- | 1.13 | 1.56 | 0.002 |
| 89 | LysoPS(22:6) | 1.53 | 570.2786 | 7 | + | C28H45NO9P+ | 0.72 | 1.26 | 0.014 |
| 90 | PS(36:1) | 9.71 | 790.5548 | 5 | + | C42H83NO10P+ | 0.86 | 1.32 | 0.019 |
| 91 | PS(42:7) | 6.04 | 879.5861 | 0 | + | C48H84N2O10P+ | 1.95 | 1.45 | 0.025 |
| 92 | PS(44:10) | 6.74 | 884.5359 | 8 | + | C50H79NO10P+ | 1.37 | 1.31 | 0.005 |
| 93 | PS(44:12) | 5.89 | 880.5045 | 8 | + | C50H75NO10P+ | 1.36 | 1.42 | 0.025 |

Statistics by Mann-Whitney *U*-test between young and old mice.

**Supplementary Table S2.** Significantly altered polar metabolites in old mice.

| Peak no. | Metabolite | RT  (min) | *m/z* | Difference (ppm) | Ionization mode | Molecular formula | Fold change (old/young) | VIP value | *p*-value |
| --- | --- | --- | --- | --- | --- | --- | --- | --- | --- |
| 1 | acylcarnitine (C5:0) | 1.18 | 246.1697 | 1 | + | C12H24NO4+ | 0.78 | 1.16 | 0.031 |
| 2 | acylcarnitine (C6:0) | 1.39 | 260.1844 | 4 | + | C13H26NO4+ | 0.72 | 1.33 | 0.031 |
| 3 | acylcarnitine (C8:0) | 1.33 | 288.2163 | 2 | + | C15H30NO4+ | 0.65 | 1.27 | 0.031 |
| 4 | N-acetyl-L-methionine | 4.26 | 190.0542 | 0 | - | C7H12NO3S- | 1.44 | 1.43 | 0.031 |
| 5 | phenylalanine | 5.73 | 164.0718 | 0 | - | C9H10NO2- | 1.40 | 1.33 | 0.023 |
| 6 | tryptophan | 5.95 | 203.083 | 1 | - | C11H11N2O2- | 1.46 | 1.53 | 0.005 |
| 7 | creatine | 7.75 | 132.0763 | 3 | + | C4H10N3O2+ | 1.37 | 1.29 | 0.042 |
| 8 | creatinine | 3.98 | 114.066 | 1 | + | C4H8N3O+ | 1.35 | 1.73 | 0.005 |
| 9 | 1-methylhistamine | 6.4 | 126.1023 | 2 | + | C6H12N3+ | 1.36 | 1.71 | 0 |
| 10 | betaine | 6.89 | 118.0855 | 6 | + | C5H12NO2+ | 0.67 | 2.28 | 0 |
| 11 | choline | 6.35 | 104.1067 | 2 | + | C5H14NO+ | 1.21 | 1.65 | 0.005 |
| 12 | deoxycholic acid | 0.87 | 391.2831 | 5 | - | C24H39O4- | 1.92 | 2.13 | 0.001 |
| 13 | taurocholic acid | 4.51 | 533.3249 | 1 | + | C26H49N2O7S+ | 1.41 | 1.14 | 0 |
| 14 | adenosine | 2.62 | 268.1036 | 1 | + | C10H14N5O4+ | 0.80 | 1.45 | 0.016 |
| 15 | hypoxanthine | 3.22 | 137.0452 | 4 | + | C5H5N4O+ | 1.13 | 1.87 | 0 |
| 16 | 5'-methylthioadenosine | 1.14 | 298.0968 | 0 | + | C11H16N5O3S+ | 1.43 | 1.96 | 0.001 |
| 17 | uracil | 1.23 | 111.0208 | 7 | - | C4H3N2O2- | 1.57 | 184 | 0.001 |
| 18 | uridine | 3.6 | 245.0755 | 5 | + | C9H13N2O6+ | 0.80 | 1.62 | 0.008 |
| 19 | uric acid | 3.71 | 167.0225 | 8 | - | C5H3N4O3- | 1.41 | 1.37 | 0.012 |
| 20 | xanthine | 3.74 | 153.0402 | 3 | + | C5H5N4O2+ | 1.42 | 1.46 | 0.042 |
| 21 | xanthosine | 4.6 | 283.0672 | 4 | - | C10H11N4O6- | 1.74 | 1.57 | 0.008 |
| 22 | p-cresyl sulfate | 0.63 | 187.0058 | 6 | - | C7H7O4S- | 8.31 | 1.54 | 0 |
| 23 | hippuric acid | 4.13 | 178.0521 | 6 | - | C9H8NO3- | 3.64 | 2.05 | 0 |
| 24 | indoxyl sulfate | 1.29 | 212.0027 | 1 | - | C8H6NO4S- | 3.67 | 2.27 | 0 |
| 25 | N-palmitoyl taurine | 0.74 | 362.2374 | 0 | - | C18H36NO4S- | 1.82 | 2.12 | 0 |
| 26 | N-stearoyl taurine | 0.73 | 390.2654 | 7 | - | C20H40NO4S- | 1.88 | 2.23 | 0 |

Statistics by Mann-Whitney *U*-test between young and old mice.

**Supplementary Table S3.** UPLC-TQ-MS MRM calibration curve equations and linear correlation coefficients (*R*^2^) for the bone tissue metabolites that were quantified.

| Compound | Con. range (ng/g) | Calibration equations | *R*^2^ |
| --- | --- | --- | --- |
| p-Cresyl sulfate | 0.1-5 | y=0.084548x+0.003132 | 0.9999 |
| Indoxyl sulfate | 5-100 | y=0.028067x-0.107158 | 0.9919 |
| Hippuric acid | 1-20 | y=0.004698x-0.002195 | 0.9977 |

**Supplementary Table S4.** UPLC-TQ-MS MRM calibration curve equations and linear correlation coefficients (*R*^2^) for the plasma metabolites that were quantified.

| Compound | Con. range (ng/g) | Calibration equations | *R*^2^ |
| --- | --- | --- | --- |
| p-Cresyl sulfate | 0.5-100 | y=0.081446x+0.004151 | 0.9996 |
| Indoxyl sulfate | 20-200 | y=0.038712x-0.613162 | 0.9955 |
| Hippuric acid | 5-100 | y=0.006899x-0.030046 | 0.9938 |

**Supplementary Table S5.** Retention times and MRM transitions of the bone tissue metabolites that were quantified by UPLC-TQ-MS.

| Compound | Retention time (min) | Ionization Mode | Precursor ion (*m/z*) | Collision energy (eV) | MRM ion transitions (*m/z*) |
| --- | --- | --- | --- | --- | --- |
| p-Cresyl sulfate | 0.86 | - | 187 | 20 | 107 |
| Indoxyl sulfate | 2.93 | - | 212 | 20 | 80 |
| Hippuric acid | 1.92 | - | 178 | 14 | 77 |


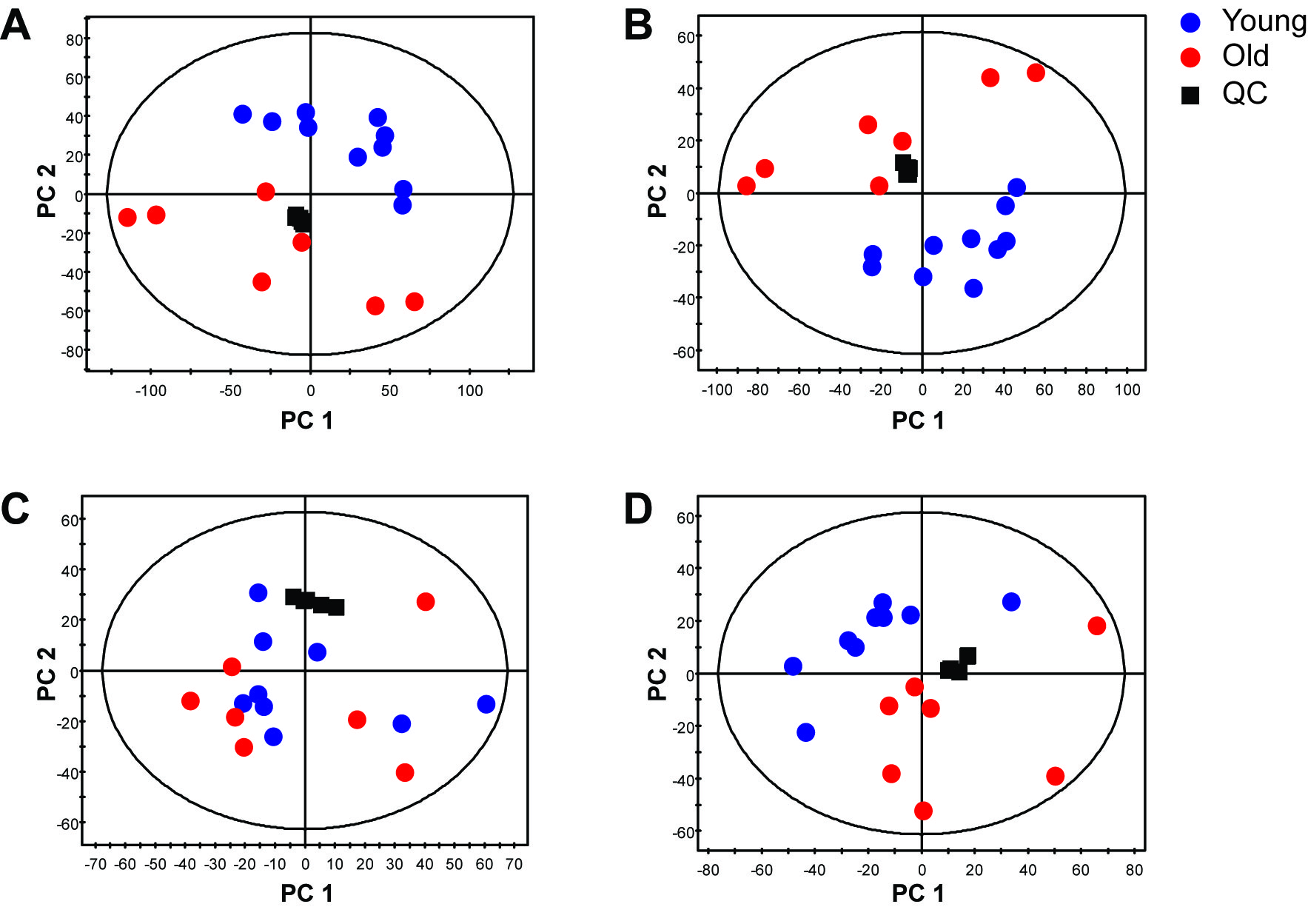


**Supplementary Fig. S1 Multivariate statistical analysis of bone lipid and polar extracts in young and old mice.** Principal component analysis (PCA) score plots from the spectra of (A) positive (R^2^X=0.574 and Q^2^=0.364) and (B) negative (R^2^X=0.536 and Q^2^=0.294) mode of UPLC-QTOF-MS in lipid metabolites and PCA score plots from the spectra of (C) positive (R^2^X=0.635 and Q^2^=0.253) and (D) negative (R^2^X=0.625 and Q^2^=0.21) mode of UPLC-QTOF-MS in polar metabolites.


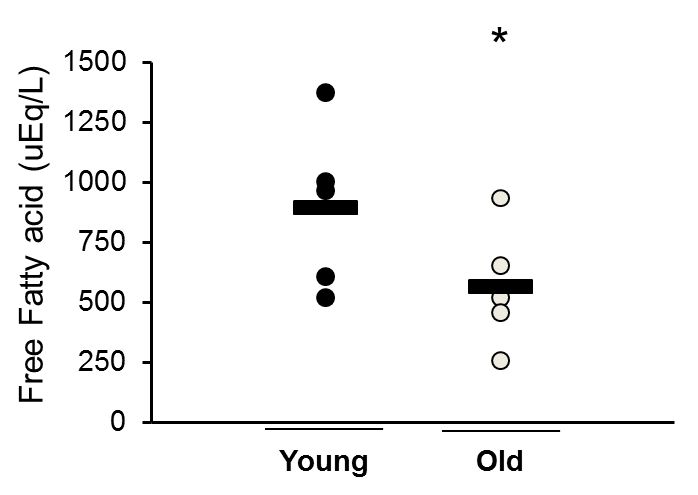


**Supplementary Fig. S2 Serum FFA levels.** FFA levels were measured in serum samples from young (closed circles) and old (open circles) mice. Statistical differences in the levels of FFA in serum of young and old mice were determined using the Mann-Whitney *U*-test. **p* < 0.05.

**
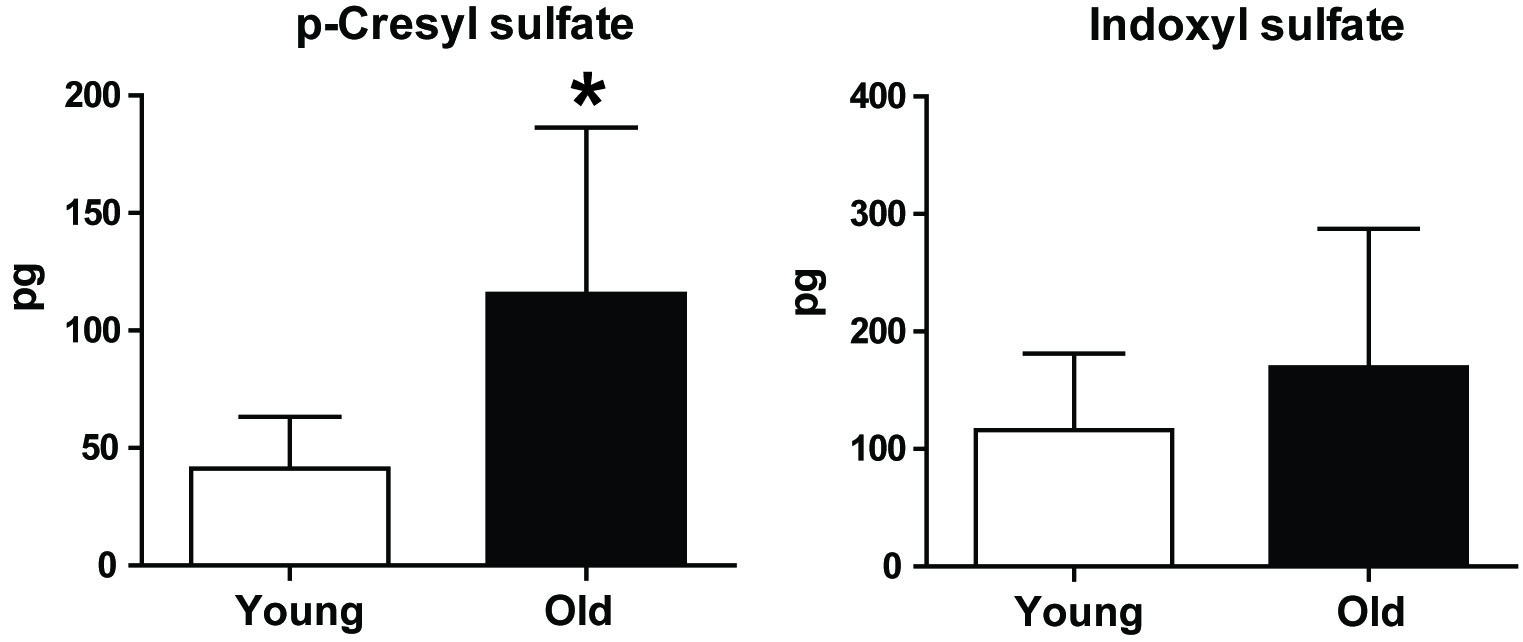
**

**Supplementary Fig. S3 Quantification of p-cresyl sulfate and indoxyl sulfate in bone marrows using UPLC-TQ-MS.** Results are expressed as mean ± SEM. Statistical differences in the levels of uremic toxin metabolites in bone marrows of young and old mice were determined using the Mann-Whitney *U*-test. **p* < 0.05.
